# A Real-Time Portable IoT System for Telework Tracking

**DOI:** 10.3389/fdgth.2021.643042

**Published:** 2021-06-10

**Authors:** Yongxin Zhang, Zheng Chen, Haoyu Tian, Koshiro Kido, Naoaki Ono, Wei Chen, Toshiyo Tamura, M. D. Altaf-Ul-Amin, Shigehiko Kanaya, Ming Huang

**Affiliations:** ^1^Computational Systems Biology, Division of Information Science, Nara Institute of Science and Technology, Nara, Japan; ^2^Data Center, Nara Institute of Science and Technology, Nara, Japan; ^3^Department of Electronic Engineering, Center for Intelligent Medical Electronics, School of Information Science and Technology, Fudan University, Shanghai, China; ^4^Institute for Healthcare Robotics, Waseda University, Tokyo, Japan

**Keywords:** telework, nudge, wearable, realtime tracking, convolutional neural network

## Abstract

Telework has become a universal working style under the background of COVID-19. With the increased time of working at home, problems, such as lack of physical activities and prolonged sedentary behavior become more prominent. In this situation, a self-managing working pattern regulation may be the most practical way to maintain worker's well-being. To this end, this paper validated the idea of using an Internet of Things (IoT) system (a smartphone and the accompanying smartwatch) to monitor the working status in real-time so as to record the working pattern and nudge the user to have a behavior change. By using the accelerometer and gyroscope enclosed in the smartwatch worn on the right wrist, nine-channel data streams of the two sensors were sent to the paired smartphone for data preprocessing, and action recognition in real time. By considering the cooperativity and orthogonality of the data streams, a shallow convolutional neural network (CNN) model was constructed to recognize the working status from a common working routine. As preliminary research, the results of the CNN model show accurate performance [5-fold cross-validation: 0.97 recall and 0.98 precision; leave-one-out validation: 0.95 recall and 0.94 precision; (support vector machine (SVM): 0.89 recall and 0.90 precision; random forest: 0.95 recall and 0.93 precision)] for the recognition of working status, suggesting the feasibility of this fully online method. Although further validation in a more realistic working scenario should be conducted for this method, this proof-of-concept study clarifies the prospect of a user-friendly online working tracking system. With a tailored working pattern guidance, this method is expected to contribute to the workers' wellness not only during the COVID-19 pandemic but also take effect in the post-COVID-19 era.

## 1. Introduction

A great leap of digital healthcare is expected under the background of COVID-19, which has changed the style of socioeconomic organization. Now, telework has been being adopted pervasively to decrease the transmission risk so as to contain the pandemic. On the other side of the strengthened working efficiency by saving the commuting time, the side effects caused by prolonged working hours become more visible.

Given that the adverse effect of prolonged working hours and the consequent stress have been identified (1, 2), attempts that chopped up the working time with breaks have been tried and validated (3, 4). Additionally, the sit-stand working pattern has been proposed recently as a new working fashion to reduce the sitting time and has shown the potential effectiveness (5). However, the change of posture may not be able to eliminate the adverse effect of prolonged working hours. Given the difficulty in keeping up to an intervention schedule by the worker himself, a fully automatic intervention system, which can recognize the working status in real-time and then generate the behavior-change notification automatically, is valuable. To meet the requirements in simultaneousness and interactiveness, the system should (1) be able to recognize the motion by extracting the information from video or time-series sensor signal in real time, and (2) be able to feedback to the subject in a convenient way.

The first requirement is widely studied in the topic of human activity recognition (HAR) by using the camera, ambient sensor, and wearable sensors (6, 7). The first two modalities are not suitable for the recognition of working status due to the higher computation cost and the rigid requirement in the system setup. Numerous previous research utilizing wearable sensor fusion in HAR show the clear prospect of accurate HAR recognition using portable sensors (8–10). Nevertheless, the aforementioned two requirements make it more difficult for most of the methods to be applied.

The smartphones and the accompanying smartwatch ecosystem, which is one of the major modalities of IoT devices, provides an excellent platform to implement the HAR in terms of performance and availability. The research of using a smartphone as the hub of data-stream acquiring, processing, and modeling have emerged in recent years. Cao et al. have tried to conceptualize the smartphone-based implementation by optimizing the number and types of features and validated the algorithm by using an open dataset (11). Cvetkovic et al. tried to fuse the accelerometer signal from a smartphone and a wristband to recognize the daily activities, which are majorly locomotions and achieved an 87% average accuracy (12). Bianchi et al. proposed a system consists of sensing and data transmitting via a wearable gadget and the accompanying HAR recognition in the form of cloud service based on the deep learning model (13). In the IoT context, the user's loyalty is an unavoidable factor in system design, which suggests that an established ecosystem, such as the iOS and Android system, with a massive user base, would be ideal. Moreover, it is natural for these commercial systems to provide a direct interaction via smartphone or smartwatch devices. With the new dedicated neural network accelerator being added in, the recent smartphones are becoming the appropriate hub for edge computing as research concerning using the signals of IMU sensor of a smartwatch can be seen lately (14, 15).

Although the monitoring of working status did not become a practical need until last year when remote working become an elementary style and few researchers contributed to the topic, human activity recognition based on wearable devices, which may be applicable, have been developed. Mannini et al. have tried to use the wrist-worn and ankle-worn accelerometer to identify the locomotions (walking, cycling, and resting) (16). Specially, as it can be imaged for a real working situation, a few relevant elementary actions, such as reading and typing, as well as irrelevant interruptions are mixed in, a coarse information, such as the record of screen time and sedentary time may cause overestimate or underestimate of working time. Therefore, Kwon and Choi have tried to construct a pipeline based on smartwatch and artificial neural network model to recognize the working relevant activities from other daily living activities (17). In considering the requirement on the accuracy and the restriction of privacy protection, the activity recognition based on wearable sensors is considered to be an appropriate solution, in comparison with the video-based, which may cause concern of the privacy disclosure and the self-report-based methods, which is prone to be coarse and data missing.

However, in the previous studies, the influence of individual difference is unclear and the overall method including signal source and the classification model can be optimized. In view of the results of the previous researches above, in this research, we extend the signal source by adding the signal of gyroscope and comparing the convolutional neural network (CNN) classification model with random forest (RF) and support vector machine (SVM) in more rigorous off-line [5-fold and leave-one-out (LOO)] and real-time experiments to evaluate the feasibility of working recognition with standalone pair of smartphone and smartwatch.

## 2. Materials and Methods

### 2.1. Data Acquisition

The data used in this research were generated by the accelerometer and gyroscopes enclosed in an Apple Watch (series 5). The coreMotion of Apple provides the application programing interface (APIs) for the developer to access the data generated by the sensors in Apple watch and iPhone, which can be extracted for off-line use or direct use in a dedicated app. Both the raw data and the processed values can be extracted by the APIs provided by coreMotion in a user-defined interval. The sampling rate is 50 Hz in this research, which is higher than 20 Hz, and it is often used in human activity recognition, because most of the target activities requires a minimal of 30 Hz to prevent aliasing (18, 19). In this study, the data streams being used in this study are tabulated in Table 1.

**Table 1:** The nine channels of data streams used in this research, which can be accessed by the APIs of CoreMotion.

**Data**	**Description**
Motionuseraccelerationx (G) Motionuseraccelerationy (G) Motionuseraccelerationz (G)	The acceleration that the user is giving to the device along the corresponding axis. The total acceleration of the device is equal to gravity plus the acceleration the user impacts to the device
Motionyaw (rad)	Angular rotation around an axis that runs vertically through the device.
Motionroll (rad)	Angular rotation around a longitudinal axis that passes through the the device from its top to Bottom
Motionpitch (rad)	Angular rotation around a lateral axis that passes through the device from side to side
Motionrotationratex (rad/s) Motionrotationratey (rad/s) Motionrotationratez (rad/s)	Rotation rate along the corresponding axis

The data streams will then be used by the coreML, which is a framework for machine learning provided by Apple. In recent models (iPhone11 and later), the computation performance is greatly boosted by the dedicated hardware—the neural engine. The machine learning model can be trained separately by python and then converted back by the coremltools to the coreML format to run on the iPhone. In this study, we extracted the data streams to train the model first, and then put the converted model back into the iPhone.

### 2.2. Experiments

In this study, we simulated the activity pattern at-home by performing the combinations of locomotion and limbs movements, which are described in Table 2. Two experiments were carried out subsequently. The first offline experiment, which also served the purpose of initial data collection, was conducted on 12 subjects (college students, 10 males, two females; ages: [23–34] years old), whose willingness in participating in the experiments was confirmed by written informed consent, with an Apple watch worn on their right wrists. The subjects were guided to conduct the prescribed locomotions and limb movements at their own paces. The timing for each action was recorded by an assistant to ensure the correctness of class labeling. In every 2 s, statistical features which is described in section 2.3 of the nine-channel signals sensed by the watch simultaneously were calculated on site and sent to the paired iPhone11 via Bluetooth. In the experiment protocol, the working-while-standing style was added to the conventional sitting style given that more and more people use at least partially the standing position in working time. The actions of reading, keyboard typing, and writing were attributed as working status. Therefore, given the similarity of the nine-channel signals, we combined limb movements of reading, typing, and writing in both standing and sitting status, which results in three classes of combinations for working status. Consequently, there are seven classes of locomotion-limbs-movements combinations to be recognized in this research as shown in the [Label] row of Table 2.

**Table 2:** The protocol of the experiment (upper) and the samples number for each class (lower).

**Episode**	**1st**	**2nd**	**3rd**	**4th**	**5th**	**6th**	**7th**	**8th**	**9th**	**10th**
Locomotion	Walking	Standing	Standing	Standing	Standing	Sitting	Sitting	Sitting	Sitting	Lying
Limb movements	Neutral	Neutral	Reading	Typing	Writing	Neutral	Reading	Typing	Writing	Neutral
Duration (min)	10	5	3	3	3	5	3	3	3	10
Label	W_N	St_N	Re	Ty	Wr	Si_N	Re	Ty	Wr	L_N
Class	W_N	St_N	Re	Ty	Wr	Si_N	L_N			
#Sample	892	429	541	539	546	444	900			
Duration (min)	119	57	72	72	73	60	120			

Finally, a real-time validation, which used the the pre-trained model to predict the simultaneous activity, was conducted. Two subjects, who have participated in the off-line experiment, were invited to repeat the experiment in order to test the repeatability of the model; another three subjects who did not participate in the offline experiment (males, 24–28 years old) were invited to perform the actions of working status for 30 min, during which time they could stop working or drinking water freely.

### 2.3. Features Extraction

Regarding the offline experiment, the nine-channel signals were stored in the iPhone during the experiment. Whereafter, signals were extracted from iPhone as CSV files with time stamps and used to train the classification model offline. Although the model can be trained directly in iPhone via Xcode, Python was used in the training period given the higher flexibility and the abundance of packages.

The preprocessing is closely related to the construction of the CNN models. Although the deep ANN is adequate in automatic feature generation, given the relatively small dataset and the effectiveness of the engineered feature extraction in reducing the input dimension and model complexity (19, 20), it is assumed that noise in a short interval (2 s) is normally distributed so that the lower order statistics features (mean and standard deviation) are sufficient in describing the short segment without filtering and the low order statistics. On the other hand, the mean and standard deviation have been proved to be informative features in activity recognition problems (19, 21). Therefore, the mean and standard deviation of the nine-channel signals were extracted from the right wrist and used as the input to the models.

During the experiment, it was noticed that the inter-subject difference in performing the writing, reading, and typing mainly resides in the pace. Therefore, a time length, which is set as 2 s, that can probably include one bout of the action is desired. This size of the window yields highest accuracy in human activity recognition, even though it includes fewer cycles of an action (18). In this logic, the means and standard deviation of a short interval (2 s) are the unit input to the CNN models.

Moreover, although it is used by default that the data are fragmented into short intervals and input into machine learning model for classification, the automatic segmentation may occur in the same semantic event (the same action), which would then worsen both the training and predicting period, especially for a relatively short 2-s window. In view of this concern, we tried to mitigate this problem by using a longer time interval of the data while keeping the basic interval as 2 s. The feature vector of each basic interval is then arranged into a row and stacked up to a feature matrix ***M*** ∈ ℜ^*m*×*n*^, where *m* represents *m* × 2 s data and *n* represents *n* features.

In addition to the mean and standard deviation, we assumed that the cooperativity of the nine channels is informative in describing the differences between the seven classes. Eventually, the features of the nine channels can be extracted in the following way:

- first, the pair-wise Pearson correlation of the nine-channel signals were calculated, which resulted in a symmetrical 9 × 9 matrix ***R***^*i*^, where the superscript represents the *i*th 2s;
- second, the maximum eigenvector ***ε***^*i*^( ∈ ℜ^1×9^) of the ***R***^*i*^ is then calculated and extracted as the features of cooperativity;
- third, the means $\mu^{i}=\mu_{1}^{i},\mu_{2}^{i},\cdots \;,\mu_{9}^{i}$ and standard deviations $\sigma^{i}=\sigma_{1}^{i},\sigma_{2}^{i},\cdots \;,\sigma_{9}^{i}$ are calculated for each channel and concatenated to ***ε***^*i*^ to form the feature vector ***ν***^*i*^ ∈ ℜ^1×27^;
- finally, the feature matrix for a longer interval is formed by stacking the feature vectors row by row to form the matrix *M* as the input of the CNN model.

**Figure 1:**
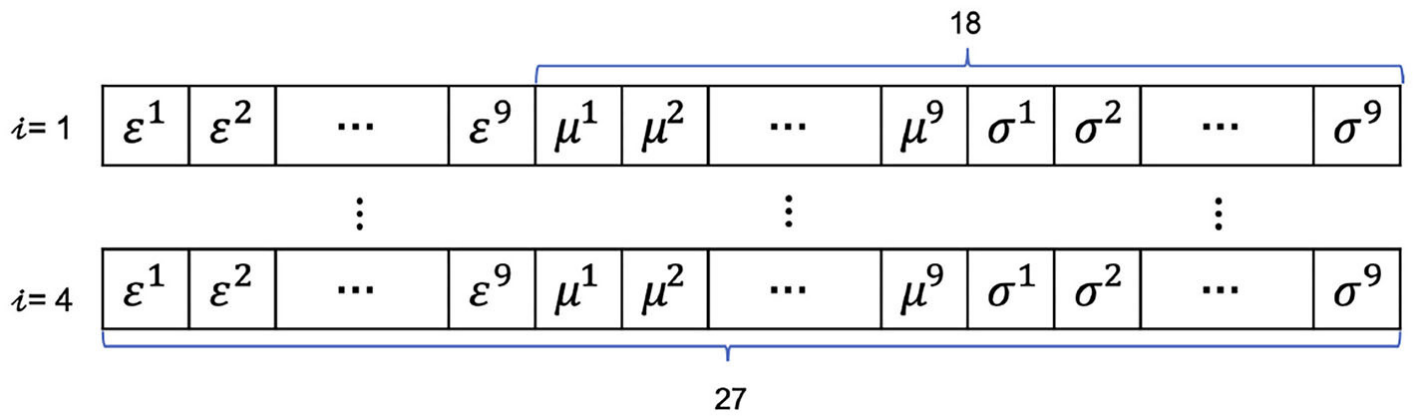
The features and the shape of model input, *i* represents the *i*th 2 s, and a sample of input consists of 8 s. The (***ε***, ***μ***, ***σ***) and (***μ***, ***σ***) features combinations are represented by the structure of 27 and 18, respectively.

The *m* and *n* are the hyperparameters, and the *m* is set as 8 s, an interval that includes most of the underlying hand movements. Regarding the *n*, the (***ε***, ***μ***, ***σ***) combination and the (***μ***, ***σ***) combination were tried in the model building stage. The shape of the input matrix is illustrated in Figure 1.

With regard to the real-time test, the nine-channels signals were sampled at 50 Hz via the **CoreMotion** framework and then were segmented into short episodes of 2-s length for further features generation by using the vDSP module in the **Accelerate** framework. On the other side, the iPhone used the Passive Response Delegate Mechanism to receive and reorganize the consecutive four 2-s feature vectors, ***f*** ∈ ℜ^*n*^, into an 8-s feature matrix ***M***, which were then fed into the pre-trained model.

### 2.4. Classification Model

**Figure 2:**
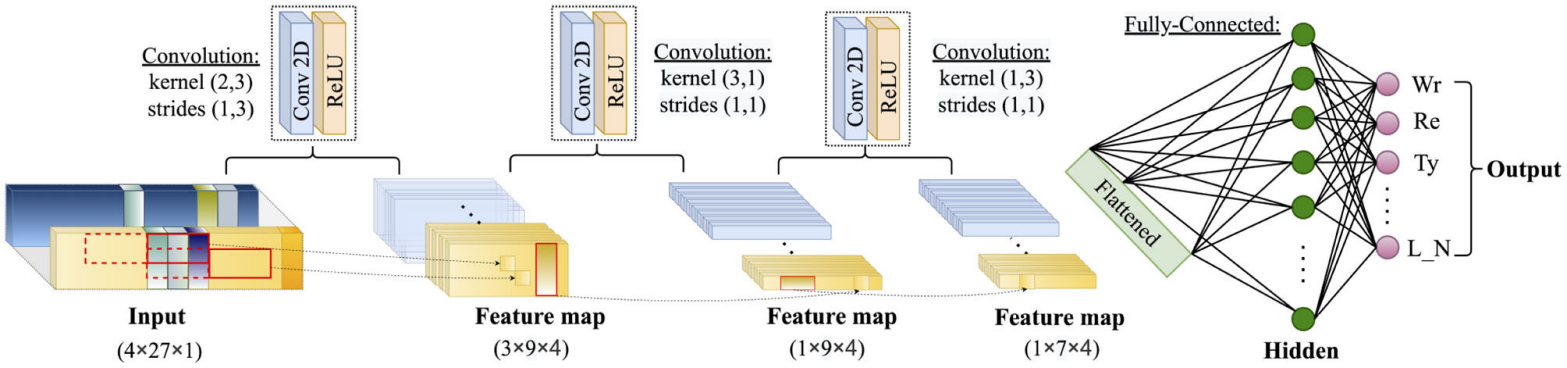
The structure of the convolutional neural network (CNN) models. The 8-s nine-channel data streams were transformed into a 4 × 27 (or 4 × 18, not shown in the figure) 2D matrix to input into the network. There are three variants, where CNN(1) uses the first convolutional layer, CNN(2) uses the first two convolutional layers, and CNN(3) uses all the three convolutional layers.

The classification model was built with a 2D CNN model with one to three hidden layers (Figure 2) in light of the finding of Münzner et al., which shows that a sallow CNN model with 2–3 CNN layers is better than the random forest model (22). The simplicity of the model also benefits the edge computation by using the iPhone at one's disposal, where the computation latency and the power consumption should be taken into account seriously. To minimize the negative influence of the deep learning model on the general performance of the smartphone, developers tend to restrict the complexity of the model (23, 24). A simpler model may also be beneficial to its generalizability and robustness when being applied to a new dataset. Figure 2 shows the structure of the CNN model, where the feature matrix ***M*** was provided as input.

As it is shown in Figure 2, in the first CNN layer, we paid special attention to the kernel size and strides, because we thought that a double length in unit interval (2 s) will be helpful in extracting the latent features of each action. This consideration also applied in the tuning of the second CNN layer, where the whole time length of the input to the second CNN layer was covered by the filters. The resultant feature map of the second CNN layer is further abstracted by the third CNN layer, which combines the features of three channels. Based on this structure, three variants were tested in the 5-fold and LOO validations, where CNN(1) corresponds to the model with the first CNN layer, CNN(2) corresponds to the model with the first two CNN layers, and CNN(3) corresponds to the model with all the three CNN layers.

With the development in machine learning, a number of classification models have been used for HAR, among which the SVM and random forest were proven to be suitable in this field (22, 25, 26). To confirm the performance of the CNN models, the SVM classifier with radial basis function (RBF) kernel (regularization parameter = 1.5) and the random forest classifier (number of trees = 200, maximum tree depth = 10), which are supported by CoreML, have been compared with the CNN models in the 5-fold and LOO validations.

### 2.5. Evaluation

Three kinds of evaluations were conducted to examine the performance of the overall pipeline. First, to examine the performance of the overall method, 5-fold cross-validation was used. The overall accuracy, F1 score and the Matthews correlation coefficient (MCC) is used to evaluate the overall performance of the model. The MCC metric is essentially a correlation coefficient, which is more suitable in evaluating the imbalanced dataset by taking the proportion of each class into consideration. The definition of MCC for the binary situation is:


(1) $$\begin{array}{l} \text{MCC}=\frac{TP\times TN-FP\times FN}{\sqrt{\left(TP+FP\right)\left(TP+FN\right)\left(TN+FP\right)\left(TN+FN\right)}}, \end{array}$$

and the MCC calculation can be easily extended to the multiclass situation. Moreover, the results for working status classification are specifically analyzed with class-wise metrics (recall, precision).

Second, because a portion of the samples from the same subject would be used in the training stage in the 5-fold validation, a validation that can evaluate the influence of the individual difference on the model is beneficial. Since the leave-one-out validation, which is more rigorous for the model generalizability by excluding the whole dataset of a subject from the training dataset and using it as the test dataset (18), it was implemented for all the 12 subjects. From this validation, we expected to determine the best model in terms of generalizability.

Finally, the real-time test, which is described in section 2.2, was finally conducted to validate the reproducibility and generalizability of the model by installing the best model into the prototype application. For the two subjects who have participated in the off-line experiment, a confusion matrix is used to show the results, whereas for the three subjects who were new to the experiment, individual compositional bar chart is used to show the detail of classification results.

## 3. Results

By using the coreMotion API, the signals of the nine channels can be extracted at a constant frequency (50 Hz). Although slight fluctuation can be seen in the sampling interval from time to time, the fluctuations were all <10 ms. Measurements were successfully extracted from all the 12 subjects, and by separating the samples for 8 s, the sample numbers of the seven classes can be found in Table 2 (lower part).

**Figure 3:**
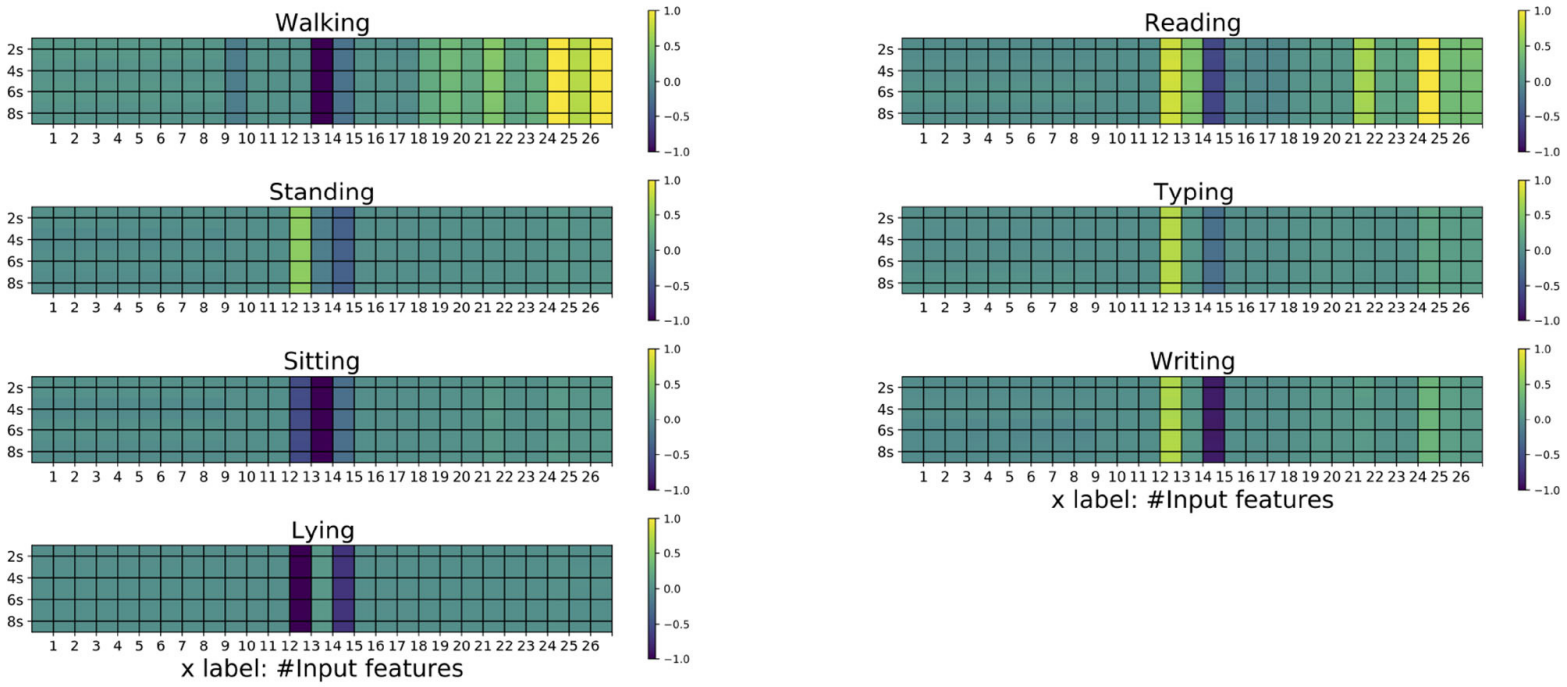
The averaged 8-s matrices for the seven classes. The matrices on the left column corresponds to *W_N, St_N, Si_N, L_N*, respectively, whereas those on the right column corresponds to *Re, Ty*, and *Wr*, respectively. The horizontal axis corresponds the maximum eigenvector of the correlation matrix, the means, standard deviation, and the of the nine channels.

By plotting the averaged 8-s matrices (Figure 3), the differences of the seven classes can be visualized. From Figure 3, the inter-classes difference can be confirmed. Specifically, the three classes that belong to the working status are similar to each other, while the walking is a distinct one being different from the others. Moreover, it can be seen that the *St_N* is similar to the *Ty*, which causes the occasional misclassification between these two classes in the 5-fold cross-validation and the leave-one-out validation.

### 3.1. Results of 5-Fold Cross-Validation

**Figure 4:**
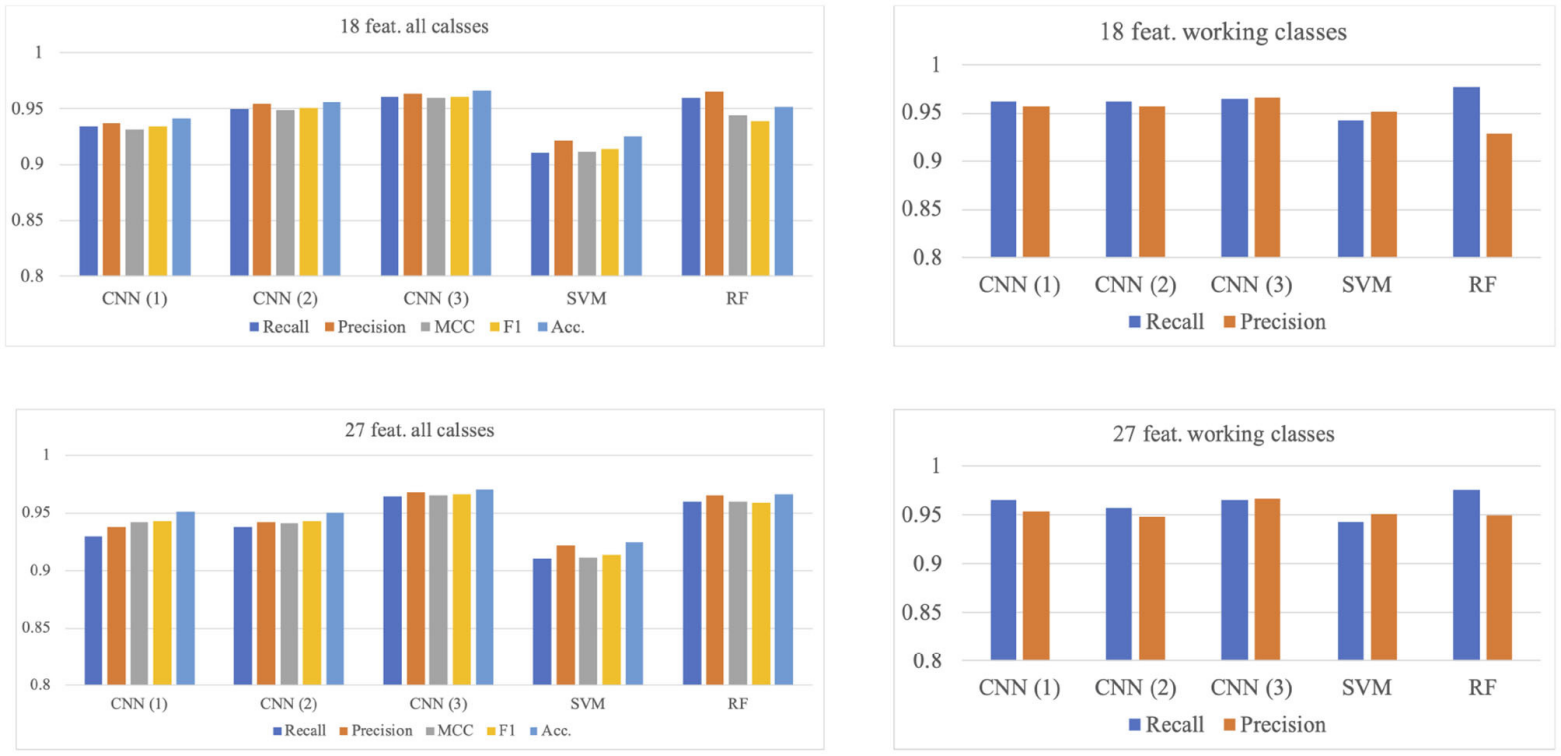
Models comparison of 5-fold cross-validation. CNN(1), CNN(2), and CNN(3) correspond to the model with 1, 2, and 3 convolutional layer(s), respectively.

**Figure 5:**
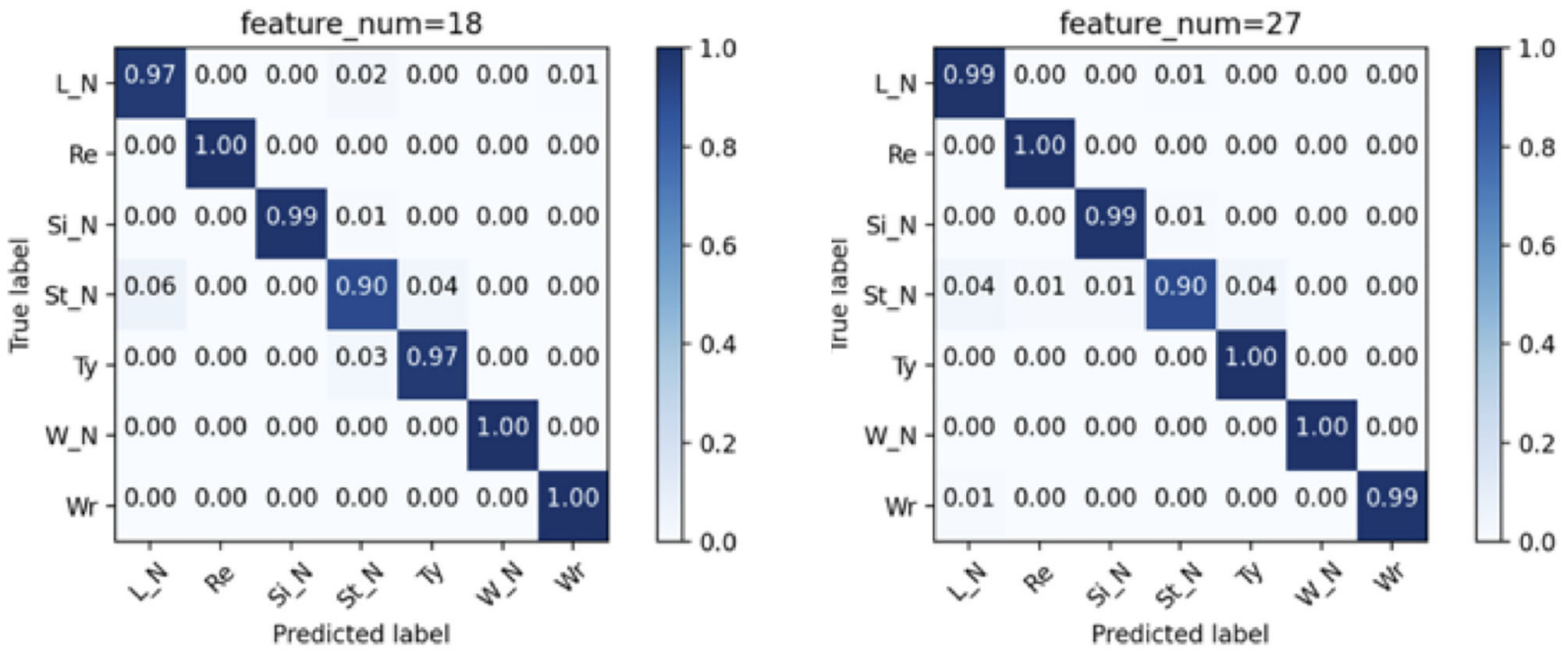
Confusion matrices of the CNN(3) model by using the two combination of features in the 5-fold cross-validation. The correspondence is indicated by the title of the matrices.

Models that used 27 features (***ε***, ***μ***, ***σ***) combination and 18 features (***μ***, ***σ***) combination are summarized in Figure 4, from which it can be confirmed that the model input with 27 features combination with three CNN layers has the best performance for the working status recognition (recall: 0.965, precision: 0.967), whose confusion matrix can be seen in the right matrix of Figure 5, while the counterpart with 18 features combination (left matrix of Figure 5) has similar results (recall: 0.964, precision: 0.965).

Looking closer into the confusion matrices of the CNN(3) models with two combination of features (Figure 5), the fluctuation in the predicting accuracy can be seen. Figure 5 shows the averaged confusion matrices of the models, from which it can be confirmed that the three actions of working status (*Re, Ty*, and *Wr*) can be recognized accurately, whereas the *St_N* is somewhat difficult to be separated from the *L_N*, which may be caused by the similarity in free movements of torso and limbs in these two actions. Within the three actions of working status, the Ty may be recognized as *St_N* occasionally, which seems plausible because the vibration of the torso while standing may also be reflected by the sensors on the right wrist.

### 3.2. Results of Leave-One-Out Validation

**Figure 6:**
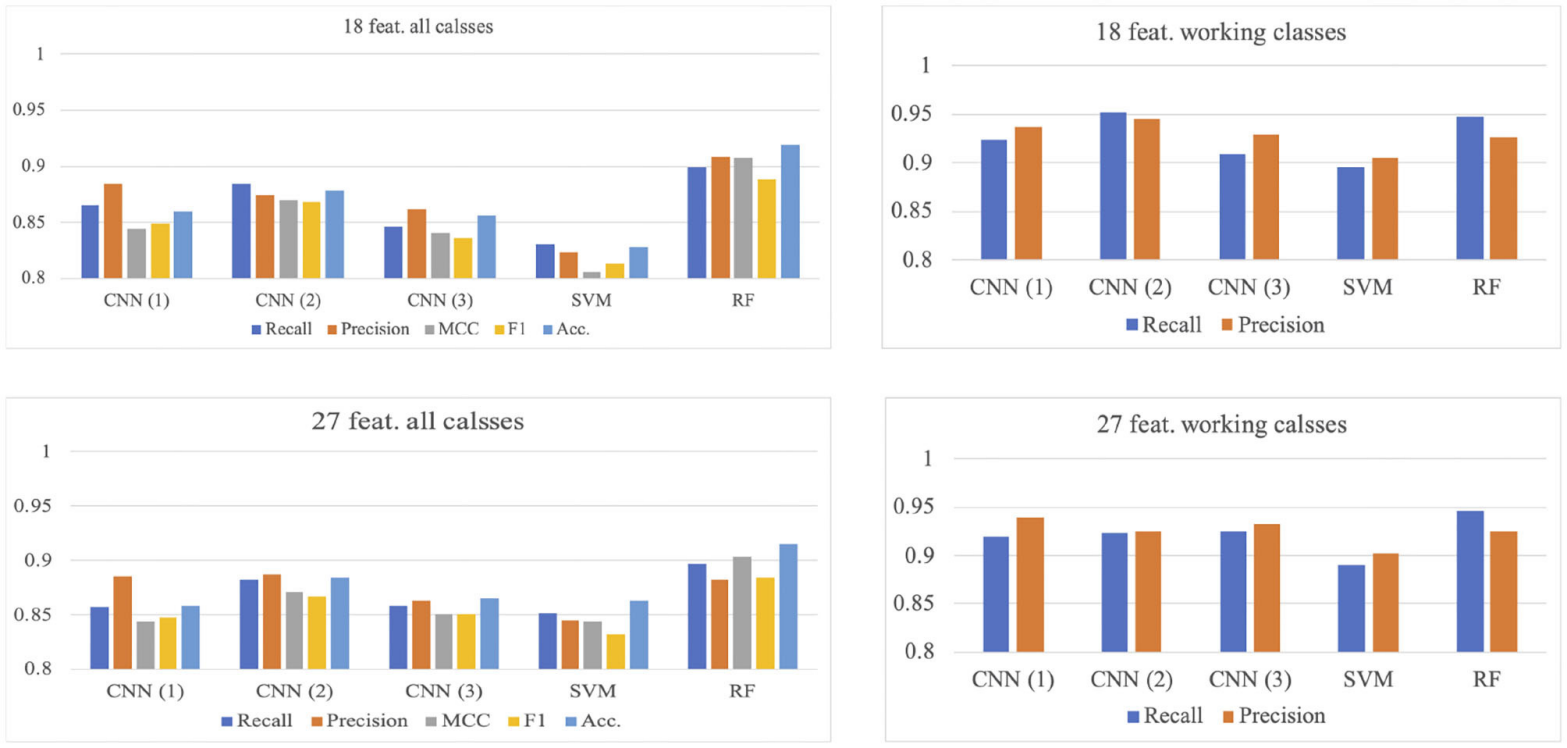
Models comparison of LOO validation. CNN(1), CNN(2), and CNN(3) corresponds to the model with 1, 2, and 3 convolutional layer(s), respectively.

Similar to the 5-fold validation, models with two feature combinations were compared here. The results are summarized in Figure 6. In this validation, the RF model is the best for the overall classification, but not for the working status. The model with two CNN layer inputs with 18 features combination has the best performance (recall: 0.951, precision: 0.944), while the RF model has a similar performance (recall: 0.947, precision: 0.926). Please note that the recall and precision values in this paragraph are the averaged values for the three working activities.

**Figure 7:**
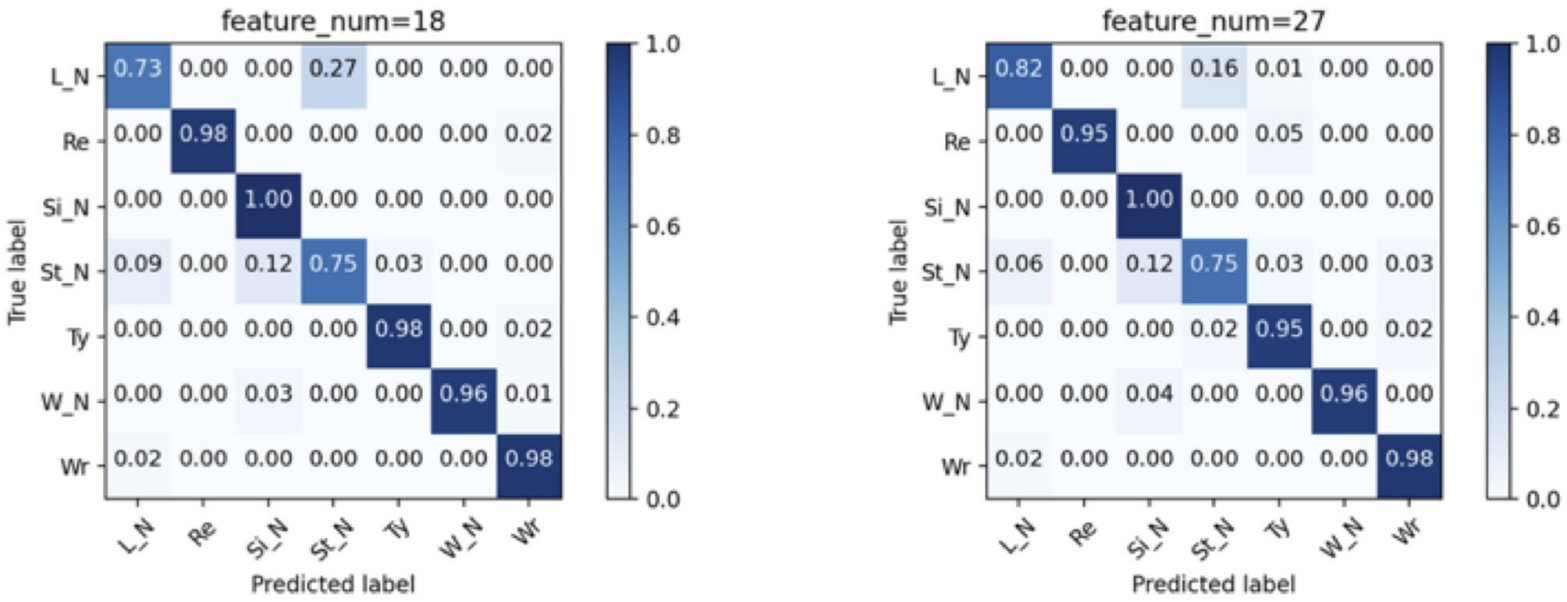
Confusion matrices of the CNN(2) model by using the two combination of features in the LOO validation. The correspondence is indicated by the title of the matrices.

The effect of different feature combinations is compared by examining 2-layers CNN model with 18 and 27 features (Figure 7). The 27-feature model outperforms the counterpart in identifying the *L_N*, but is inferior in identifying the *Re* and *Ty*. For both models, the *St_N* cannot be classified accurately, for being misclassified as *L_N* and *Si_N*. This inaccuracy is accountable; given that the subjects are free to move their hands occasionally, there is no consistent pattern for these three classes.

**Figure 8:**
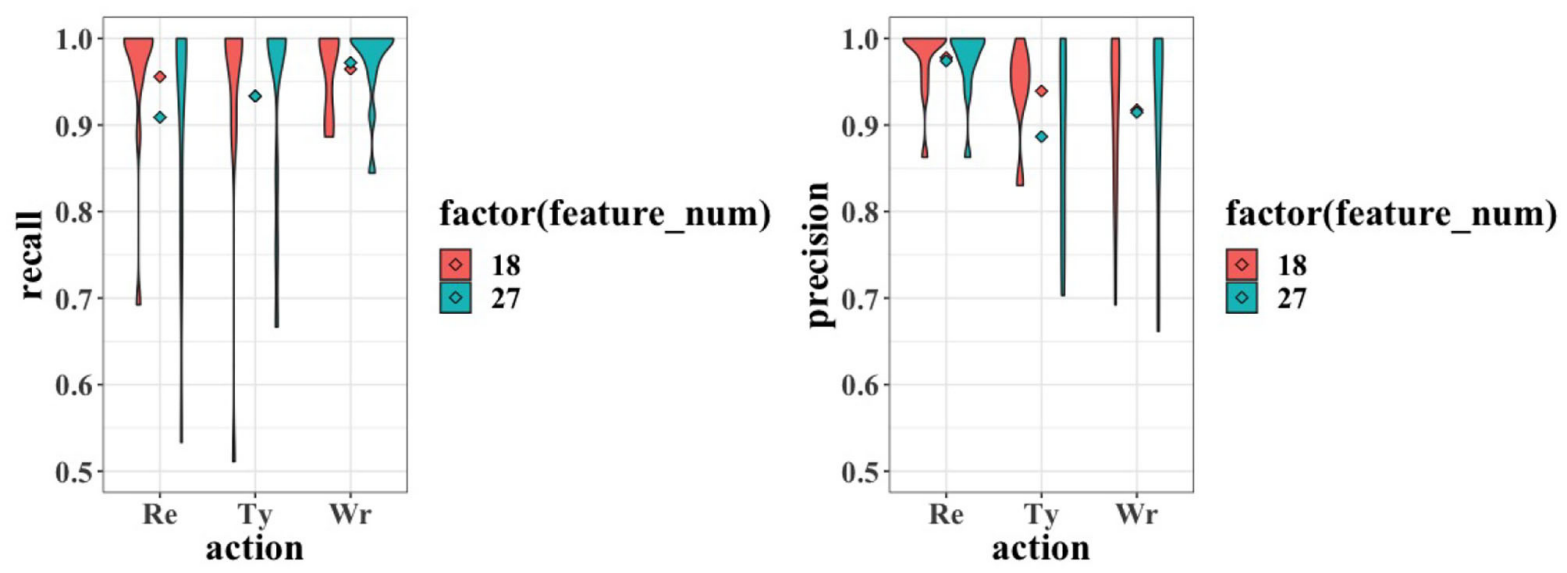
Violin plot for the recall **(left)** and precision **(right)** values of the working status. Each violin shows the distribution of the corresponding metric over the LOO validation, whereas the dots in the middle of each pair of violins show their means.

Figure 8 further shows the distribution of the recall and precision over the 12 validations. The long-tail distribution can be seen in the 27-feature model for *Re* and the 18-feature model for *Ty*. However, they are caused by only one case (subject) with extremely low result, respectively. Therefore, no severe overfitting is observed.

### 3.3. Results of Real-Time Validation

**Figure 9:**
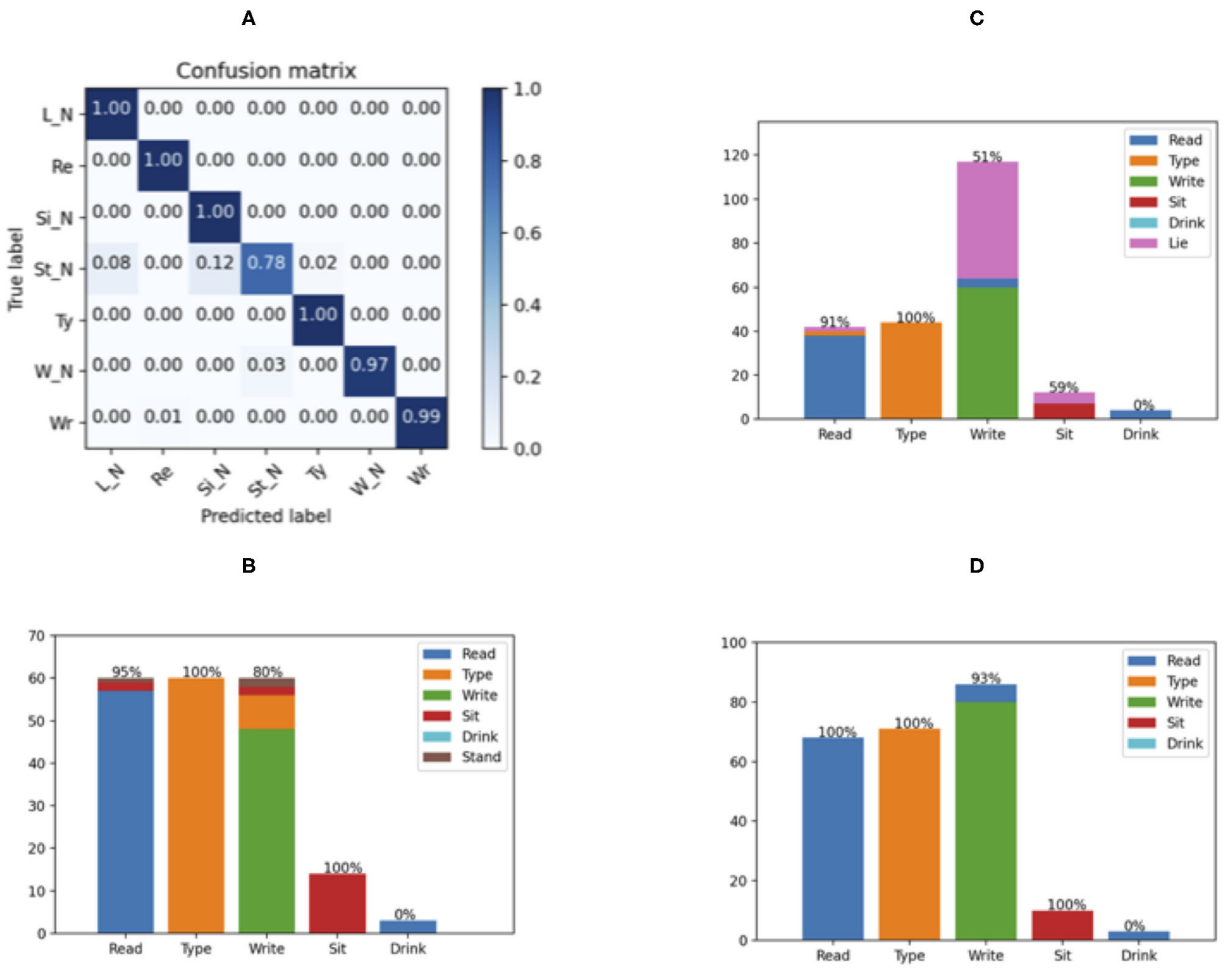
Results of the real-time validation. The confusion matrix of the 2 subjects who have participated in the off-line experiment is shown in **(A)**; whereas **(B–D)** show the compositional bar chart of each new subject. The values on top of each bar represent the ratio of correct recognition for each classes.

According to the results of the offline LOO validations, the 18-feature two-layers CNN model achieved the best results for working status recognition. Therefore, it is implemented to the prototype application in iPhone and tested by real-time experiments. The averaged confusion matrix of the two subjects who have participated in the offline experiment is shown in Figure 9A, whereas the Matrix in Figures 9B–D shows the compositional bar chart of the three new subjects.

According to Figure 8 , the results of the two subjects who have participated in the offline experiment are consistent with the 5-fold validation, having a generally accurate classification.

The results for the three new subjects vary mainly in the recognition of writing, while the reading and typing activities can be recognized accurately. The writing was recognized as typing in Figure 9B and as lying in Figure 9C. This variance may be caused by the unconstrained action of the hand, whereas the reading and typing require the hand to interplay with the book and keyboard. Noteworthily, the new subjects were free to interrupt their activities and perform irrelevant actions, such as drinking water, which made it more similar to the real working situation. While the sitting can be recognized, the drinking is mistakenly classified as reading, probably due to the similarity in hand movement. Albeit the misclassification of these random actions irrelevant to the working status, the majorities of the three working activities have been recognized correctly. A simple statistical threshold can output a correct judge whether the user is working or not.

## 4. Discussion

This proof-of-concept study focuses on the availability of the overall method of combining wearable sensing and edge computing based on the iOS ecosystem. Therefore, the usefulness of the wrist-worn sensors, the selection of the preprocessing scheme, and the performance of a complexity-restricted machine-learning model are the three major factors. First, the performance of the CNN model confirms the feasibility of working status recognition by using the wrist-worn sensors in the Apple Watch alone, where all the three actions that belong to the working status can be recognized accurately. Although more atomic actions, i.e., drinking water, can be added to the model, judging from the results of the classification, it is plausible to expect a similar outcome for the working status recognition.

### 4.1. Pre-processing

The selection of preprocessing can be reflected in the generation of input vectors. From the comparison of the two combinations, it can be seen that the cooperativity between channels expressed by the channel-wise linear correlation is not as important as the low-order statistical features (the mean and standard deviation). This piece of insight is beneficial for the edge computing because a major part of computation in the preprocessing is for the calculation of cooperativity.

### 4.2. The CNN Model

Consistent with the results of Münzner et al. a shallow neural network is sufficient in activities recognition, this research attains the best results for LOO validation with a two-layer CNN model, a similar result as that of Münzner et al.'s research (22). By comparing the CNN models with different layers (1–3) and adding the spontaneous gyroscope signal, further improves the performance of a classification model in working status recognition without using the GPS information, which may cause misclassification of the activities that are irrelevant to the working status (17).

**Figure 10:**
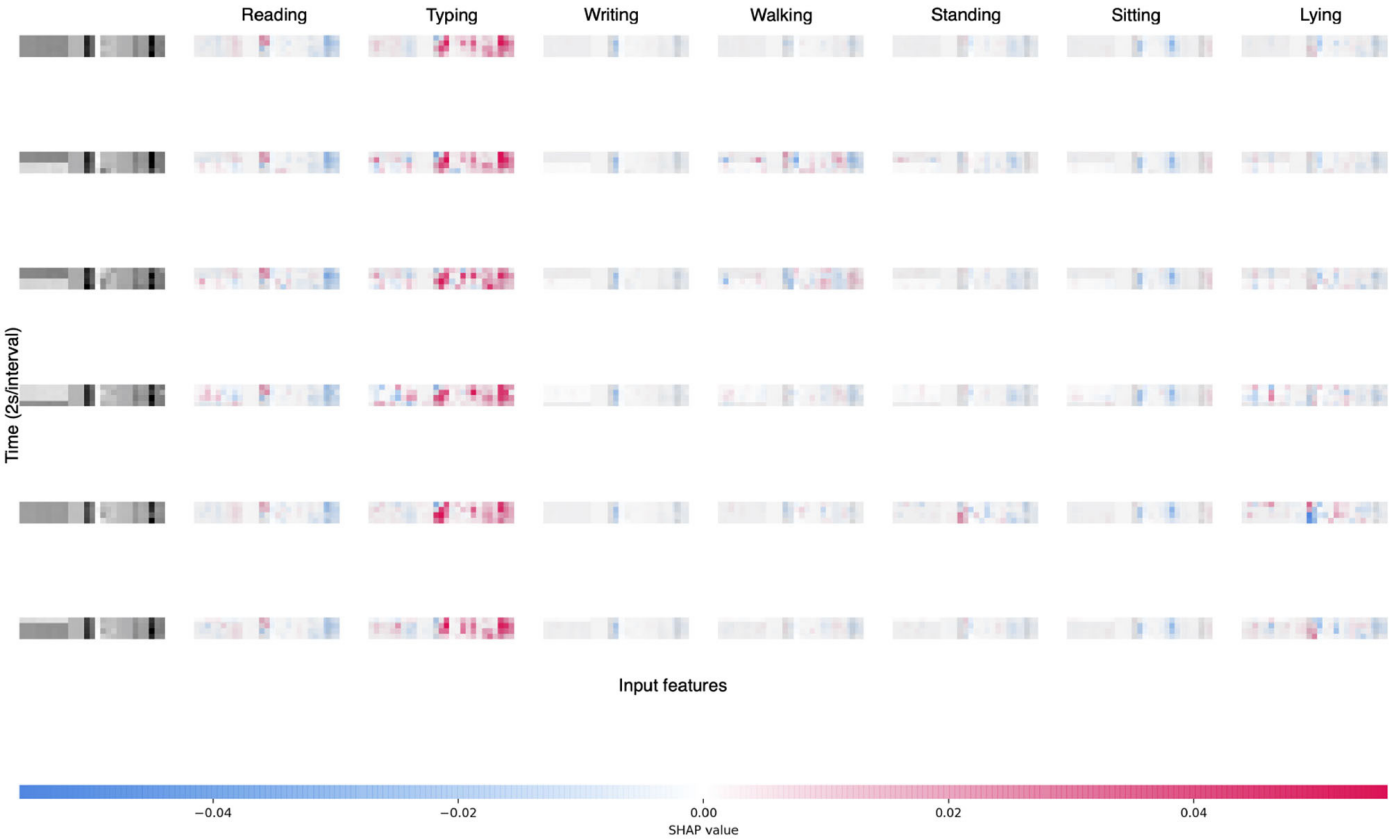
SHAP values of six samples of different classes. The subfigures in the first column are the inputs of the samples, and the subfigures from the second to the last columns represent the seven classes in the order of L_N, Re, Si_N, St_N, Ty, W_N, Wr.

Along with the model explanation by using SHAP values, the importance of the features and their special patterns for each class can be inspected closer. Figure 10 plots the SHAP values of six randomly selected samples of different classes. The subfigures from the second to the last columns represent the seven classes. First, for these six samples, the cooperativity features in the first nine columns of each subfigure have generally small SHAP values compared with the statistical features, which goes along well with the comparing results of the two different inputs. Second, although the samples are of different classes, the patterns are relatively constant for each class. For example, the subfigures in the third column correspond to the *Re* action. Although the classes of the samples vary, the patterns of the feature importance (a large absolute SHAP value implies an important feature) are very similar concentrating on the latter part of the feature vectors.

### 4.3. Limitations

We have to admit that the experiment setting is not as flexible as the real scenario, where the continuity of each action cannot be assured; by further summarizing these atomic actions into semantic features, a more realistic working pattern could be recognized using the current hardware setup. Furthermore, as it can be seen in the real-time validation, new subjects may deteriorate the model accuracy, further extension of the dataset, and further training of the model with transfer learning could be considered in future works.

### 4.4. Prospect

Coming back to the ultimate purpose of this research, the usefulness and availability of the system are equally important. This premise drives us to point at the established ecosystem, where the APIs for data acquisition and modeling are expected to be further improved. Not only this working status recognition but detailed behavior pattern modeling for all-day routine and lifestyle-physiological outcome association can also be expected in the future research.

## 5. Conclusion

Aiming at providing a timely nudging to mitigate the minus effect of long-time telework without an additional device, this research examines the idea of using wrist-worn sensors in a commercial smartwatch and a smartphone to capture the real-time signals from sensors and conduct the recognition using a pre-trained CNN model. In this manner, the whole workflow can be implemented in real time with a ready hardware setup. On the other hand, by taking the power consumption of smartphone computing into account, shallow CNN structure with special consideration on the properties of the signal is validated. By rearranging the statistical features of an 8 s signal into a feature matrix and input it into the classification model, the CNN model show accurate performance [5-fold cross-validation: 0.97 recall and 0.98 precision; leave-one-out validation: 0.95 recall and 0.94 precision (SVM: 0.89 recall and 0.90 precision; random forest: 0.95 recall and 0.93 precision)] for the recognition of working status. This proof-of-concept study clarifies the prospect of a user-friendly online working tracking system, which recognizes the working status with standalone pair of smartphone and smartwatch and will nudge the user to take a break after a long working time. It is expected to contribute to the workers' wellness not only during the COVID-19 pandemic but also take effect in the post-COVID-19 era.

## Data Availability Statement

The raw data supporting the conclusions of this article will be made available by the authors, without undue reservation.

## Ethics Statement

The studies involving human participants were reviewed and approved by Ethics Review Committee concerning Research involving Human Subjects of Nara Institute of Science and Technology. The patients/participants provided their written informed consent to participate in this study.

## Author Contributions

All authors listed have made a substantial, direct and intellectual contribution to the work, and approved it for publication.

## Conflict of Interest

The authors declare that the research was conducted in the absence of any commercial or financial relationships that could be construed as a potential conflict of interest.
